# Forensic validation of a panel of 12 SNPs for identification of Mongolian wolf and dog

**DOI:** 10.1038/s41598-020-70225-5

**Published:** 2020-08-06

**Authors:** Hong Hui Jiang, Bo Li, Yue Ma, Su Ying Bai, Thomas D. Dahmer, Adrian Linacre, Yan Chun Xu

**Affiliations:** 1 College of Wildlife and Protected Areas, Northeast Forestry University, No. 26, Hexing Road, Xiangfang District, Harbin, 150040 China; 2 National Forestry and Grassland Administration Research Center of Engineering Technology for Wildlife Conservation and Utilization, No. 26, Hexing Road, Xiangfang District, Harbin, 150040 China; 3 National Forestry and Grassland Administration Detecting Center of Wildlife, No. 26, Hexing Road, Xiangfang District, Harbin, 150040 China; 4 Ecosystems Ltd, Hong Kong, China; 5 College of Science and Engineering, Flinders University, Adelaide, SA 5042 Australia

**Keywords:** Biological techniques, Ecology, Genetics, Zoology

## Abstract

Wolf (*Canis lupus*) is a species included in appendices of CITES and is often encountered in cases of alleged poaching and trafficking of their products. When such crimes are suspected, those involved may attempt to evade legal action by claiming that the animals involved are domestic dogs (*C. l. familiaris*). To respond effectively to such claims, law enforcement agencies require reliable and robust methods to distinguish wolves from dogs. Reported molecular genetic methods are either unreliable (mitogenome sequence based), or operationally cumbersome and require much DNA (un-multiplexed microsatellites), or financially expensive (genome wide SNP genotyping). We report on the validation of a panel of 12 ancestral informative single nucleotide polymorphism (SNP) markers for discriminating wolves from dogs. A SNaPshot multiplex genotyping system was developed for the panel, and 97 Mongolian wolves (*C. l. chanco*) and 108 domestic dogs were used for validation. Results showed this panel had high genotyping success (0.991), reproducibility (1.00) and origin assignment accuracy (0.97 ± 0.05 for dogs and 1.00 ± 0.03 for wolves). Species-specificity testing suggested strong tolerance to DNA contamination across species, except for Canidae. The minimum DNA required for reliable genotyping was 6.25 pg/μl. The method and established gene frequency database are available to support identification of wolves and dogs by law enforcement agencies.

## Introduction

Grey wolf (*Canis lupus*) was once abundant over much of Eurasia and North America. However, some regional populations have suffered a severe decline in numbers because of habitat destruction and human interference^1–3^, poaching^4,5^, and human-wolf conflicts^6,7^. Although the global population is stable^8^, the Convention on International Trade in Endangered Species of Wild Fauna and Flora (CITES) list grey wolf in Appendix I (populations of Bhutan, India, Nepal and Pakistan) and Appendix II (other populations)^9^. The objective of these listings is to restrict international trade in wolves and wolf body parts. The species is also protected by national laws of most range countries.

Culturally, wolf bodies and body parts are often believed to have magical powers^10^ and medicinal functions^11–15^. Such unfounded superstitions as well as human-wolf conflicts are factors that drive poaching and trafficking of wolves and body parts in Eurasian countries. For example, media reports from China include at least 35 trafficking cases uncovered by China customs from 2010 to 2018. Seizures included hundreds of skins, carcasses, mounted specimens, and more than a thousand carpal bones and canines being trafficked from Mongolia, Russia, Kazakhstan, and Kyrgyzstan. In attempts to avoid penalties, traders often claimed that the bones and canines were those of large dogs and were sold as counterfeits of wolf body parts. Prosecution of these cases is dependent on definitive subspecies identification.

There are many morphological differences between wolves and dogs. The most apparent differentiation is based on cranial morphology^16,17^. However, studies on morphological diagnostic characters of other body parts may not result in accurate species identification. DNA-based identification offers a potentially reliable solution to this problem. Current genetic evidence indicates that dog and wolf share a common ancestry as recently as 27,000 years ago^18^. Domestication led to dogs differentiating gradually from wolves to a level of subspecies, and so named *C. l. familiaris*^19^, but their gene pools exhibit great homogeneity and contain detectable historical and recent genetic admixtures^20–24^. For this reason, coding regions of the mitochondrial genome have either no resolution between wolf and dog (12S and 16S ribosomal RNA genes)^25,26^, or detectable but poor resolution (Cyt b and COI genes)^27^. The control region (CR) holds a large number of polymorphic sites in the neutrally evolving hypervariable regions (HV)^28,29^, where some separation of wolf versus dog have been successful^30,31^. However, identification based on CR sequence may not be reliable unless additional nuclear markers are combined^32,33^ especially when the reference sample size is not sufficient to represent total genetic variation^34^.

In contrast to these sequence-based mitochondrial markers, allelic frequency-based microsatellite markers have been tested and proved effective in this regard. For instance, six autosomal microsatellites were adequate to distinguish Scandinavian wolves from dogs^35^, and 12 microsatellites achieved a very high power of identification^33^. To date, numerous microsatellites have been isolated from the canine genome and subsequently characterized and multiplexed^36–39^. The results have become a rich and convenient resource for forensic identification of wolf and dog.

SNPs are alternative nuclear markers to microsatellites for population genetic analysis, but often show higher tolerance to DNA degradation and lower analysis cost^40^. Based on next generation sequencing technology, massive genotyping of SNPs is now possible^41,42^, allowing deep detection of genetic relatedness between populations. For example, Pilot et al. found widespread and long‐term admixture between grey wolves and domestic dogs across Eurasia by analyzing 61 k SNPs^21^. Ancestry-informative SNP markers are SNPs that exhibit substantially different frequencies between various populations. A panel of ancestry-informative SNP markers can be used to estimate the proportion of ancestry of an individual wolf or dog derived from each population^43,44^. These studies highlight the potential of ancestry-informative SNP markers to separate closely related subspecies such as wolf and dog.

In order to provide an effective method for forensic identification of wolves and dogs by using advantages of SNPs assay, we randomly selected 13 dog-wolf ancestry-informative SNP markers identified in vonHoldt et al.^43^ and Zhang et al.^44^ and characterized them in samples collected from the Mongolian wolf and domestic dog using a SNaPshot multiplex assay. This system was then validated for forensic identification of the two subspecies following the Society for Wildlife Forensic Science (SWFS) Standards and Guidelines for Wildlife Forensic Analysis—Version 3^45^.

## Results

### Repeatability of genotyping

**Figure 1:**
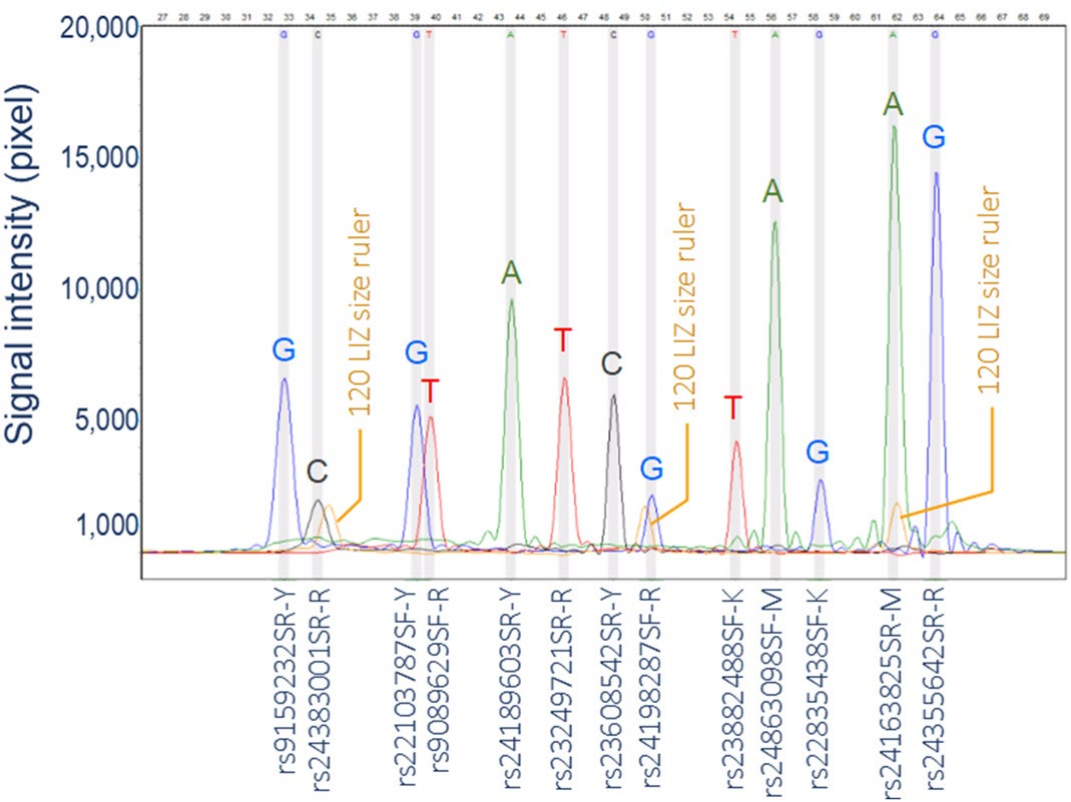
Electropherogram of a wolf sample genotyped using the multiplex SNaPshot of 13 loci. Color letters are alleles called for the signals. Letters Y, K, R and M in the name of extension primers are degenerate codes for nucleotide options at SNP sites: Y = C/T, K = G/T, R = A/G, M = A/C.

All SNP loci in the panel generated clear allele signals. Although the intensity of the signals varied from 1,000 to 40,000 pixels (Fig. 1), all alleles were called without ambiguity. However, the success of one-time genotyping of rs22103787 was 0.926 for wolf but as low as 0.444 for dog (Table 1). As a result, this locus was discarded from the panel. For the remaining 12 SNP loci, the average success of one-time genotyping was 0.990 for wolf (0.938–1.000) and 0.993 for dog (0.972–1.000). The overall average success of one-time genotyping was 0.991 for the two subspecies. The average cost to genotype one sample was around US$ 8.60.

**Table 1:** One-time genotyping success of the panel of 13 SNPs in wolf and dog samples.

SNP loci	Mongolian wolf (n = 97)	Domestic dog (n = 108)	Overall (n = 205)
rs22103787*	0.926	0.444	0.673
rs22835438	1.000	0.991	0.995
rs23249721	1.000	1.000	1.000
rs23608542	0.969	0.991	0.980
rs23882488	1.000	0.991	0.995
rs24163825	0.990	1.000	0.995
rs24189603	1.000	0.972	0.985
rs24198287	1.000	1.000	1.000
rs24355642	0.938	1.000	0.971
rs24383001	0.979	1.000	0.990
rs24863098	1.000	1.000	1.000
rs9089629	1.000	0.991	0.995
rs9159232	1.000	0.981	0.990
Average*	**0.990**	**0.993**	**0.991**

*Locus rs22103787 was excluded due to its poor genotyping success for domestic dog.

The success of genotyping was also tested on samples of different quality. The proportion of one-time successfully genotyped loci varied from: 0.83 (10/12, n = 2) to 0.92 (11/12, n = 3) for skins in high states of decay (n = 3) and viscera (n = 2); from 0.92 (11/12) to 1.00 (12/12) for moderately decayed skins (n = 3) and muscles (n = 2); and 1.00 (12/12) for samples in early stages of decay. One-time genotyping results between visceral samples (highly decayed) and skin samples (moderately decayed) were the same (0.92).

### Characterization of SNP markers

Only two alleles were observed for each SNP marker for both wolf and dog (Table 2). The difference in frequencies between the two alleles ranged from 0.848 to 1.000 across all loci for wolf, with an average of 0.939 ± 0.061. The difference ranged from 0.281 to 0.856 and averaged 0.656 ± 0.197 for dog. For either allele on a locus, the difference of frequency between wolf and dog populations ranged from 0.640 to 0.889 with a mean of 0.797 ± 0.080.

**Table 2:** Characteristics of 12 SNP markers in wolf and dog populations.

SNP loci	Wolf (n = 80)					Dog (n = 90)					Frequency difference of either allele between wolf and dog
	Genotype & frequency			Allele & frequency		Genotype & frequency			Allele & frequency		
rs22835438	GG	GT	TT	G	T	GG	GT	TT	G	T	
	1.000	0.000	0.000	1.000	0.000	0.056	0.111	0.833	0.111	0.889	0.889
rs23249721	CC	CT	TT	C	T	CC	CT	TT	C	T	
	0.000	0.038	0.962	0.019	0.981	0.756	0.167	0.078	0.839	0.161	0.820
rs23608542	GG	GA	AA	G	A	GG	GA	AA	G	A	
	0.886	0.089	0.025	0.930	0.070	0.033	0.167	0.800	0.117	0.883	0.814
rs23882488	GG	GT	TT	G	T	GG	GT	TT	G	T	
	0.000	0.025	0.975	0.013	0.988	0.552	0.310	0.138	0.707	0.293	0.694
rs24163825	GG	GT	TT	G	T	GG	GT	TT	G	T	
	0.000	0.113	0.888	0.056	0.944	0.878	0.100	0.022	0.928	0.072	0.872
rs24189603	GG	GA	AA	G	A	GG	GA	AA	G	A	
	0.000	0.152	0.848	0.076	0.924	0.878	0.100	0.022	0.928	0.072	0.852
rs24198287	GG	GA	AA	G	A	GG	GA	AA	G	A	
	0.855	0.145	0.000	0.928	0.072	0.025	0.100	0.875	0.075	0.925	0.853
rs24355642	CC	CT	TT	C	T	CC	CT	TT	C	T	
	1.000	0.000	0.000	1.000	0.000	0.090	0.169	0.742	0.174	0.826	0.826
rs24383001	CC	CT	TT	C	T	CC	CT	TT	C	T	
	0.950	0.050	0.000	0.975	0.025	0.022	0.247	0.730	0.146	0.854	0.829
rs24863098	CC	CA	AA	C	A	CC	CA	AA	C	A	
	0.000	0.000	1.000	0.000	1.000	0.483	0.315	0.202	0.640	0.360	0.640
rs9089629	GG	GA	AA	G	A	GG	GA	AA	G	A	
	0.925	0.075	0.000	0.963	0.038	0.044	0.244	0.711	0.167	0.833	0.796
rs9159232	GG	GA	AA	G	A	GG	GA	AA	G	A	
	1.000	0.000	0.000	1.000	0.000	0.180	0.270	0.551	0.315	0.685	0.685

### Assignment accuracy

**Figure 2:**
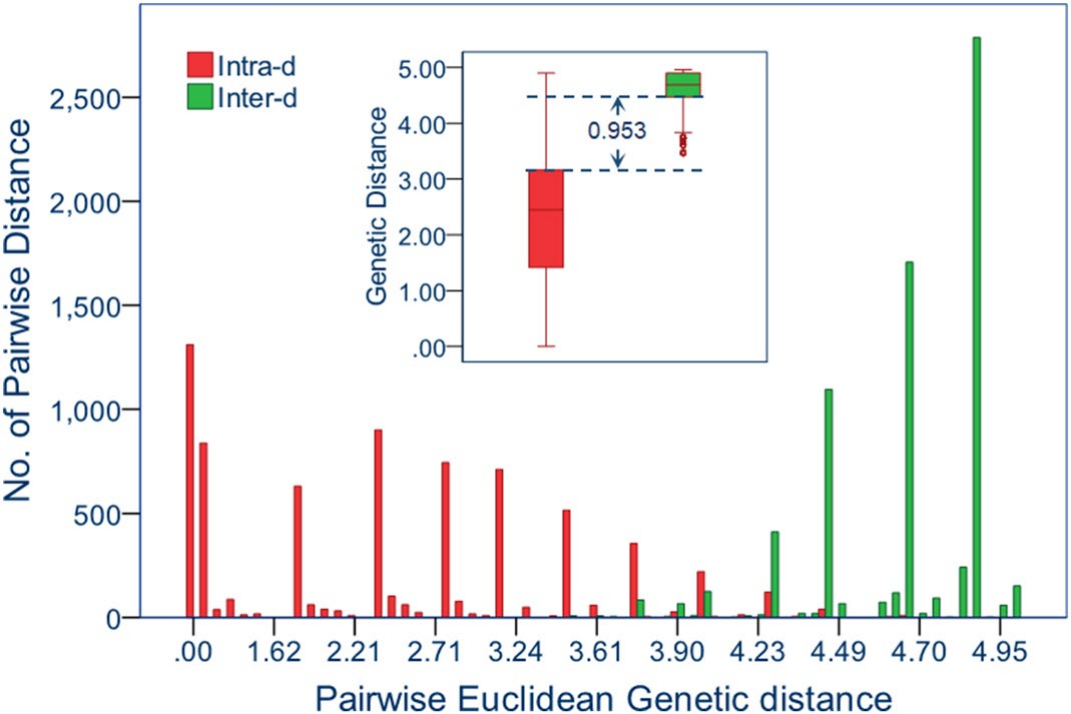
Frequency distribution of intra- and inter-subspecific pairwise Euclidean genetic distance (Intra-*d* and inter-*d*) for wolves and dogs inferred from the panel of 12 SNPs.

Euclidean pairwise genetic distance among wolves ranged from 0 to 3.464 with a mean of 1.197 ± 1.066. This range was slightly smaller than that among dogs, which ranged from 0 to 4.899 and averaged 2.980 ± 0.776. The overall intra-subspecific distance was 2.194 ± 1.274. Inter-subspecific distances ranged from 3.464 to 4.959 and averaged 4.686 ± 0.265, significantly greater than intra-subspecific distances (*t* = 162.552, *p* = 0.000). Histograms of the frequency distributions of intra-subspecific distances overlapped slightly with those of inter-subspecific distances (Fig. 2). The difference between the upper limit of intra-*d* and the lower limit of inter-*d* at 95% confidence was 0.953.

**Figure 3:**
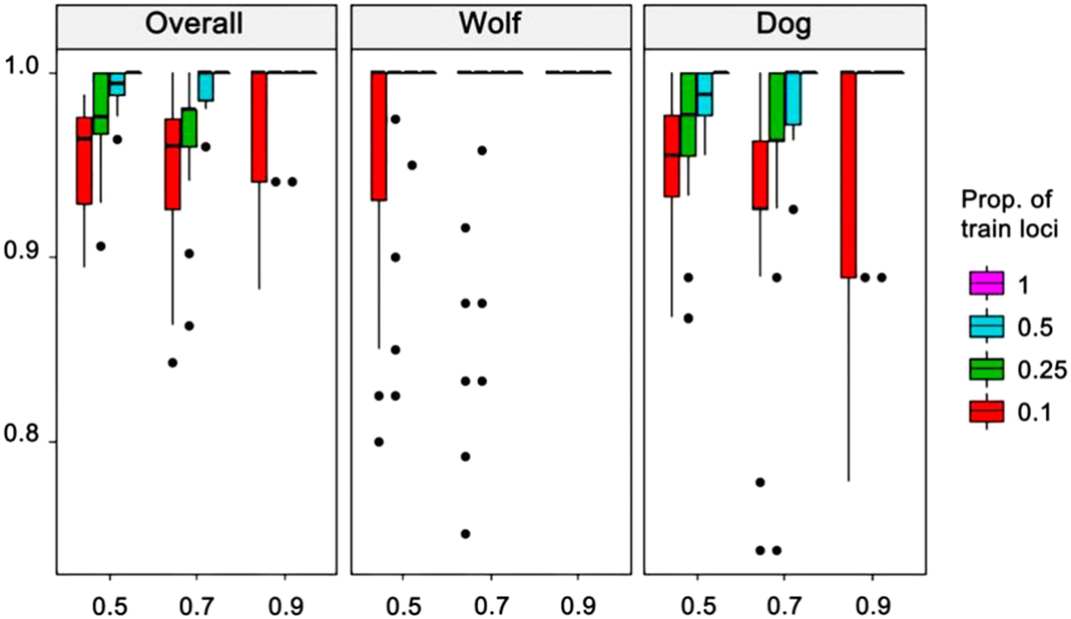
Assignment accuracies estimated via Monte-Carlo cross-validation. Proportions of individuals used for training were 50%, 70% and 90% from each population (*x*-axis); Four proportions of loci used for training were the highest 10%, 25% and 50% of *F*_st_ loci and all loci (color-coded boxes).

Assignment accuracy was estimated via Monte-Carlo cross-validation with three proportion levels of training individuals (50%, 70% and 90% of individuals from each population) crossed by four proportion levels of training loci (the highest 10%, 25% and 50% *F*_st_ loci and all loci). Results showed that this panel of SNPs had great accuracy for wolves when only the 25% highest *F*_st_ loci were used for the training that included 50% of individuals. However, similar accuracy could not be achieved unless all 12 loci were applied for 50% and greater proportions of individuals included in training for domestic dogs (Fig. 3). When the whole panel of SNP markers was used, the probabilities of wolf and dog being assigned correctly to their original groups reached 0.99 ± 0.04 and 0.97 ± 0.04, respectively.

Furthermore, K-fold cross-validation showed that when all 12 SNP loci were used, the membership probability of all wolves and dogs reached nearly 100% for K = 3, 4 and 5 (Fig. S1). All SNP markers were verified 48 times, and the membership probability of domestic dogs being correctly assigned to their subspecies was 0.97 ± 0.05. The membership probability of Mongolian wolves being correctly assigned to their subspecies was as high as 1.00 ± 0.03, providing good supports the use of the assay in forensic practice.

### Blind test with known samples

Blind tests were performed using 17 wolves and 18 dogs. All animals were assigned to their original species (17/17 and 18/18). The membership probability of tested dogs being identified as dogs was 1.000 and that for wolf incorrectly identified as dog was 4.978 × 10^–28^ with standard deviation (SD) 2.112 × 10^–27^. The membership probability of tested wolves belonging to wolf was 0.9999997 (± 1.023 × 10^–6^), and that of wolf being incorrectly identified as dog was 2.833 × 10^–7^ (± 1.023 × 10^–6^). These data suggest that the probabilities of correct assignment for wolves and dogs were about 10^7^–10^28^ greater than those of incorrect assignment.

### Reproducibility

Reproducibility of the SNaPshot method was tested using 2 wolves and 1 dog using the same protocol repeated 10 times on three different PCR machines. Results showed that all samples were successfully genotyped and the genotypes of each sample at 12 SNP loci were in complete accordance between each repeat and on different PCR machines.

### Species-specificity

Genotyping of the SNP panel yielded negative results in 9 of 12 species including human, brown bear, African lion, tiger, sika deer, red deer, moose, Siberian roe deer, and domestic cattle. For the two canid species most closely related to dogs and wolves (Arctic fox and red fox) positive allele signals were detected at 8 loci (66.7% of all loci). Wild boar was the only prey species that showed positive allele signals. Specifically, five loci showed clear positive allele signals, two loci showed negative results, and five loci showed ambiguous signals that differed between two repeated efforts at genotyping (Table 3).

**Table 3:** Genotyping results of 12 species across the panel of SNPs*.

Species	rs22835438	rs23249721	rs23608542	rs23882488	rs24163825	rs24189603	rs24198287	rs24355642	rs24383001	rs24863098	rs9089629	rs9159232
*Vulpes lagopus* (n = 8)	+	+	+	+	+	−	−	−	+	−	+	+
*V. vulpes* (n = 2)	−	+	+	+	+	−	−	−	+	+	+	+
*Ursus arctos* (n = 1)	−	−	−	−	−	−	−	−	−	−	−	−
*Panthera leo* (n = 2)	−	−	−	−	−	−	−	−	−	−	−	−
*P. tigris* (n = 2)	−	−	−	−	−	−	−	−	−	−	−	−
*Sus scrofa* (n = 1)	+	−	+	±	−	+	±	+	±	±	±	+
*Cervus nippon* (n = 2)	−	−	−	−	−	−	−	−	−	−	−	−
*C. elaphus* (n = 2)	−	−	−	−	−	−	−	−	−	−	−	−
*Alces alces* (n = 1)	−	−	−	−	−	−	−	−	−	−	−	−
*Capreolus pygargus* (n = 1)	−	−	−	−	−	−	−	−	−	−	−	−
*Bos taurus* (n = 1)	−	−	−	−	−	−	−	−	−	−	−	−
*Homo sapiens* (n = 4)	−	−	−	−	−	−	−	−	−	−	−	−

* ± Stands for contradicting genotype between two repeated attempts at genotyping.

### Sensitivity

**Figure 4:**
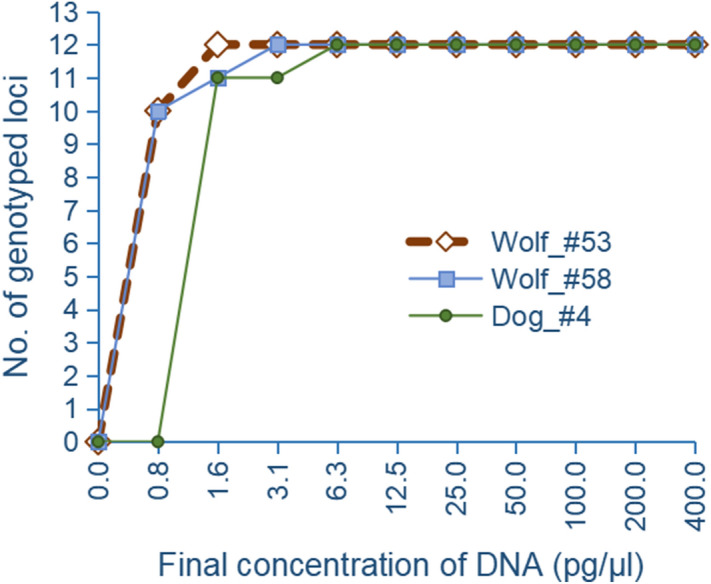
Percent of successfully genotyped loci in the SNPs panel at serial concentration input of wolf and dog total DNA.

Sensitivity test showed that the whole panel of 12 SNP loci was successfully genotyped for all three samples when the final concentration of total DNA in the reaction system reached 6.25 pg/μl. One dog locus failed to genotype when DNA concentration was reduced to 3.125 pg/μl. This did not occur for wolf until DNA concentration further was reduced to 1.560 pg/μl (Fig. 4). This suggested the minimum concentration of total DNA input to guarantee genotyping in this system was 6.25 pg/μl.

## Discussion

Speciation of dog from wolf under natural and artificial selection has led to specific genetic combinations from the ancestral gene pool for favored phenotypes^46–48^. This process produced numerous ancestral informative SNP markers that are highly informative to differentiate wolves from dogs^43,44^. However, they cannot be incorporated into for forensic practice without systematic validation being performed; this complies with the SWFS Standards and Guidelines for Wildlife Forensic Analysis^45^. For this purpose, we set up and validated a panel of 12 such SNPs, which generated reproducible and reliable SNP data from a range of sample types, such as highly decayed skins, suitable for incorporation in to forensic practices.

The reproducibility of the genetic markers is essential in forensic practice. Because all SNP loci in this panel were diallelic and showed significantly biased allelic frequency to either wolf or dog (Table 4), each provided differential information and combination of the 12 loci reached a high accumulative discriminant power. Our analysis demonstrated a gap spanning 0.953 between the upper limit of intra-*d* and the lower limit of inter-*d* at 95% confidence, indicating a low probability of false positive subspecies assignment. Cross validation showed the assignment accuracy of the whole panel reached 0.99 ± 0.04 for wolf and 0.97 ± 0.04 for dog (Fig. 3), and membership probability nearly reached 100% for all wolves and dogs (Fig. S1). Empirical (blind) tests further showed 100% apparent assignment correctness and the membership probabilities of correct assignment were 10^7^–10^28^ times greater than those of incorrect assignment. These figures suggest that this panel of SNPs has robust discriminative power between wolf and dog. An individual with the correct assignment membership probabilities fall between 10^7^–10^28^ could be expected as dog-wolf hybrid.

**Table 4:** Primers for multiplex amplification of 13 SNP markers.

Locus	Name*	Sequence (5′-3′)	T_m_	Amplicon size (bp)
rs22103787	rs22103787F	CAAGGAGGGGCTCAACCTCA	64.86	225
	rs22103787R	AGAAGGGGTGTGTTGGTTTCTACA	63.12	
rs22835438	rs22835438F	GGGATTGAACCCCTGACCTGAGT	66.77	163
	rs22835438R	TGAACCATGACCACCCCTCGT	67.10	
rs23249721	rs23249721F	TGAAATGCTCTCCGGCTGAGGT	67.58	170
	rs23249721R	TCCATGCTAAATGTCCCAGGAG	63.44	
rs23608542	rs23608542F	ACTTCTCCACGCGGAAACACTT	64.44	143
	rs23608542R	GAAATGGAAGGTGGCATTAGAACC	63.99	
rs23882488	rs23882488F	GTCCTAGTCCTGGTCCATAGAAGAGC	63.87	183
	rs23882488R	AAGTCCGAGAAAAGTGGAGGATAGAT	62.89	
rs24163825	rs24163825F	GCTAGTCCTATGCCCTGAAACTCAT	63.13	161
	rs24163825R	TCCTTATGAGCCTTAGTTTTGCTACCT	62.86	
rs24189603	rs24189603F	GGAGCTCGGAGTGACTATACTGGAAT	64.17	92
	rs24189603R	CCTCGGATGCAGTCTGTTTCTCTG	66.63	
rs24198287	rs24198287F	GTAGGCCAGTCTTCAGTGTGGTGA	64.91	323
	rs24198287R	GGCATGATTCCTGGTGGTTTTC	64.85	
rs24355642	rs24355642F	CTCATAAAATCGGGAGCCTTGCTT	65.66	105
	rs24355642R	GCAGTATACACAGCAATCCAGCAGA	64.75	
rs24383001	rs24383001F	TACGTCTGACGCCAAAACCCACT	67.02	120
	rs24383001R	CACCTGCACATTTGGGCCACT	67.61	
rs24863098	rs24863098F	GTGAGCAGAGTTGCATGGCACA	67.01	147
	rs24863098R	GCTGCTGCCTCTCAAGGACGA	67.54	
rs9089629	rs9089629F	TGGATTTGTTGCCTCTCCCCTAGT	66.17	147
	rs9089629R	CTACACTGTAAATGGGCAGCTACATCC	65.00	
rs9159232	rs9159232F	TCCCACCTACATCTGCTTCTGTGT	64.33	169
	rs9159232R	GGGATGACTTGTCAAGGAACTGAGAC	65.53	

*The letter F/R stands for forward and reverse primer.

The second but important concern in forensic practice is the robustness of operation. SNaPshot technology is a single-base extension assay with capillary electrophoresis as its detection system. It offers the advantage of easy integration into operational forensic laboratories without the requirement for any additional equipment^51^. We chose to use SNaPshot technology for the 12 loci with the intention to allow easy utilization of this panel. Allele signals of all loci were sufficiently clear to designate alleles without ambiguity. Empirical tests on various platforms obtained 100% reproducibility and repeatability. The overall average success of one-time genotyping reached 0.991 with an average cost US$ 8.6 per sample. These results demonstrate that experimental analysis of this panel is robust, reliable and cost effectively.

The ability to generate genetic data from highly compromised samples, as often encountered in wildlife forensic science, is essential such that the resulting data are not prone to false assignment^52–56^. Ideal amplified fragment size proposed for SNaPshot assay ranges from 60 to 100 bp^57^. The amplified fragment size we designed for this panel varied between 92 and 323 bp with an average 160.3 bp. This range is not much different from other PCR-based forensic genotyping systems^58–60^, though not reduced to below 100 bp. Our tests on highly decayed samples showed the proportion of one-time successfully genotyped loci greater than 0.83. The sensitivity of normal quality sample was as low as 6.25 pg/μl. These data indicate that this SNP panel is trustable to genotype most evidence if it is not extremely degraded.

Specificity of markers is a double-edged issue for wildlife forensic science. Low specificity is conducive to cross-species utilization^61,62^, but on the other hand, is prone to genotyping errors raised by DNA contamination^63^. Therefore, the specificity test here was performed with a view on contamination issues. We observed that this panel did not yield positive genotyping results in human and species of Ursidae, Felidae, Cervidae, and Bovidae, but did generate a genotype from two Canidae species, Arctic fox and red fox at 8 loci. Five loci unexpectedly yielded allele signals from a sample taken from a wild boar of Suidae (Table 5). These results suggest DNA contamination from Canidae and Suidae species may raise a risk of incorrect exclusion. Even though these species are distantly related to wolf and dog in comparison to the closeness between wolf and dog, and the membership probabilities of these contaminants might be extremely low, our data could not determine the risk based on the present sample size. Therefore, caution should be exercised when this method is applied to scenarios such as bite identification of Canidae and Suidae victims. Fortunately, discriminating wolves from dogs is often required after evidence is assigned to wolf-dog group by routine species identification, for example, using cytochrome b^27^.

**Table 5:** Extension primers for genotyping of 13 SNP markers.

Name*	Sequences	T_m_	Expected size (bp)
rs22103787SF-Y	(T)_7_-CCTCTCCTCTCCTTTAGTTCAACA	60.28	32
rs22835438SF-K	(T)_27_-GGCGTCATGGTATATCTGTAAATCAG	62.00	54
rs23249721SR-R	(T)_13_-CCCAGAATTTGAGATAACAAACATCA	62.44	40
rs23608542SR-Y	(T)_17_-AAAATGTTAGCGTAGAGCCGTTATT	60.69	43
rs23882488SF-K	(T)_24_-AAATAGCTCATTTGCCCAGATATG	63.13	49
rs24163825SR-M	(T)_31_-GCTACCTATTTGGTGAGAATAGTAAT	59.11	58
rs24189603SR-Y	(T)_12_-GTTTCTCTGTAGCCCTGTTTGTCAC	62.54	38
rs24198287SF-R	(T)_17_-CTTCATCAGTTACAAATAATTGTGGTG	60.10	45
rs24355642SR-R	(T)_31_-CAGTAACTTCCCTTCGTTTAATTAGTCTAC	60.52	62
rs24383001SR-R	(T)_2_-CTTGGAACCGAGAATGAAGAGC	62.46	25
rs24863098SF-M	(T)_26_-GGAGTGCATAATGATCCCTGTGTAC	62.94	52
rs9089629SF-R	(T)_9_-AACCACTGGATTACTGCCATTGA	62.82	33
rs9159232SR-Y	GCGTCCCTGTGACCTAGTTTC	61.06	22

*F and R stand for forward and reverse primer; letters Y, K, R and M behind the short dash—are degenerate codes for nucleotide options at SNP sites: Y = C/T, K = G/T, R = A/G, M = A/C.

We note that the reference wolves in this study represented populations in north and northeast China, Mongolia, and the Russian Far East, while reference dogs were all sampled from China. The assignment accuracy should be tested for wolves and dogs outside these regions before this method is more widely applied.

In conclusion, the panel of the 12 SNPs are highly effective for forensic discriminating wolves from dogs with high assignment accuracy. The SNaPshot multiplex system had high genotyping success, reproducibility, sensitivity and tolerance to poor DNA quality. Species-specificity is adequate to avoid DNA contamination from taxa except for Canidae, thus this method should be used only if the evidence is identified as from a wolf-dog group. The established gene frequency database in this study are ready to support forensic identification of wolves and dogs by law enforcement agencies.

## Materials and methods

### Samples collection

Naturally dried skin and fresh blood samples of 97 Mongolian wolves (*Canis lupus chanco*) were collected from museums, wildlife safari parks, and zoos with their kind permission. Ninety-six of these wolves were born in the wild of north and northeast China, Mongolia and the Russian Far East. The remaining wolf was born in captivity to parents captured in northeast China. The taxonomy of these wolves was verified according to morphology and breeding records prior to sample collection. We also sampled a total of 108 large dogs, including German shepherd, border collie, mongrel, golden retriever, American Eskimo, and mongrels. For each dog, a stain of K_2_-EDTA-anticoagulated blood on sterilized gauze was obtained from pet hospitals after receipt of consent from dog owners, dried naturally and preserved at 4 °C for 1 to 20 days until DNA extraction. Eighty wolves and 90 dogs were randomly chosen to set reference groups, and 17 wolves and 18 dogs were used for blind testing.

A total of 15 dog and Mongolian wolf samples from different tissues with different quality were collected for the purpose of a repeatability test of sample in different quality. This comprised: 8 skin samples (slightly decayed, n = 5; moderately decayed, n = 3), 5 muscle samples (moderately decayed, n = 2; highly decayed, n = 3) and 2 viscera samples (highly decayed). All these samples were collected from museums where they were preserved at − 20 °C in refrigerators for years.

Samples were also collected for species specificity testing, including human (*Homo sapiens*, hairs, n = 4), canid species, viz. Arctic fox (*Vulpes lagopus*, frozen semen, n = 8) and red fox (*Vulpes vulpes*, frozen semen, n = 2), brown bear (*Ursus arctos*, muscle, n = 1), African lion (*Panthera leo*, muscle, n = 2), tiger (*Panthera tigris*, muscle, n = 2), and prey species including wild boar (*Sus scrofa*, muscle, n = 1), sika deer (*Cervus nippon*, muscle, n = 2), red deer (*Cervus elaphus*, muscle, n = 2), moose (*Alces alces*, muscle, n = 1), Siberian roe deer (*Capreolus pygargus*, muscle, n = 1), and cattle (*Bos taurus*, muscle, n = 1). Human hair samples were donated by volunteers in our team with the informed consent of all the participants. All other animal samples were provided by the State Forestry and Grassland Administration Detecting Center of Wildlife. All sample collection and experimentation were approved by Northeast Forestry University Institutional Review Board of Ethics and Administration of Experimental Animals (Approval No. 2018001).

### SNP genotyping

Total DNA was isolated from each sample using AxyPrep Genomic DNA Isolation Kit (Kang Ning Life Science Ltd., China). DNA was quantified using NanoPhotometer (Implen, Germany) and diluted to 10 ng/μl. Thirteen dog-wolf ancestry-informative SNP markers were selected from Zhang et al. (2014), viz. rs9159232, rs22835438, rs23249721, rs24863098, rs23608542, rs23882488, rs9089629, rs24198287, rs24189603, rs24163825, rs24383001, rs24355642 and rs22103787^44,62^. Sequence information for these SNP markers is listed in Supplement Information Table S1. All 13 of these ancestry-informative SNP markers revealed great divergence of allelic frequency between wolf and dog in previous tests^43,44^. Primer pairs were designed based on these sequences (Table S1) for multiplex amplification of fragments, each containing an SNP site (Table 4). The size of amplified fragments ranged from 92 to 323 bp.

All SNP loci were genotyped using the SNaPshot Multiplex System (Applied Biosystems, USA). Multiplex PCR of all 13 markers was set up in a 20 μl system containing 1 × HotStarTaq buffer, 3.0 mM Mg^2+^, 0.3 mM dNTP, 1 U HotStarTaq polymerase (Qiagen Inc., Germany) and 1 μl total DNA. Final concentration of each pair of primers was 0.05 µM except for rs24163825F/R and rs24355642F/R of which final concentrations were 0.025 µM. Cycling program included 1 cycle at 95 °C for 2 min; 11 cycles at 94 °C for 20 s, 65 °C for 40 s, and 72 °C for 1.5 min; 24 cycles at 94 °C for 20 s, 59 °C for 30 s and 72 °C for 1.5 min; followed by 72 °C for 2 min and 4 °C forever.

Five unit of SAP enzyme (Progema) and 2U Exonuclease I (Epicentre) were added to 10 μl of PCR products. The mixture was incubated at 37 °C for 30 min and then inactivated at 75 °C for 15 min. Amplicons were purified using High Pure PCR Product Purification Kit (Roche Diagnostics Ltd., China). An SNP extension reaction was set up in a 10 μl system containing 5 μl SNaPshot Multiplex Ready Reaction Mix (Applied Biosystems), 2 μl purified multiplex PCR amplicons, 1 μl extension primers mix (0.8 μM of each primer, Table 5) and 2 μl deionized water. Extension reaction was performed using the program: 1 cycle at 96 °C for 1 min; 28 cycles at 96 °C for 10 s, 55 °C for 5 s, and 60 °C for 30 s; followed by 4 °C forever. After cycling was finished, 1 U SAP was added to the reaction system and incubated at 37 °C for 1 h followed by inactivation at 75 °C for 15 min. Subsequently, 0.5 μl of extension product was transferred to a new tube, mixed with 0.5 μl Liz120 size standard and 9 μl Hi-Di (Applied Biosystems), denatured at 95 °C for 5 min, and separated on ABI3730XL DNA analyzer (Applied Biosystems). GeneMapper 4.1 Software (Applied Biosystems) was used to read genotypes of all SNPs. Individuals with failure at one or two loci were re-genotyped by adjusting T_m_ and template concentration until the genotype was complete.

### Separation of pairwise genetic distance

Pairwise Euclidean distances within and between subspecies were calculated based on SNP genotype data of the reference populations using PopGen in R^62^. The distribution of frequency at each distance interval was visualized using a histogram that was built using SPSS 19.0 (IBM SPSS Statistics, Chicago, USA). 95% confidence intervals (CIs) were computed for within- and between-subspecific distances. The safety of subspecies assignment was then estimated using the difference between the lower bound of 95% CIs for inter-subspecific distances (inter-*d*) and the upper bound of 95% CIs for intra-subspecific distances (intra-*d*).

### Resampling cross-validation

Assignment accuracy was tested using two resampling cross-validations performed using the assignPOP package in R^63^. Two hypothetical populations were defined using reference samples of wolf (n = 80) and dog (n = 90). F_st_ value of each locus was calculated by the assignPOP package in R. Monte-Carlo cross-validation was performed with 50%, 70%, and 90% of randomly selected individuals from each population crossed by the top 10%, 25%, and 50% of high F_st_ loci, and all loci as training data. Linear discriminant analysis was performed to build predictive models. Each combination of training data was resampled 30 times, for a total of 360 assignment tests (30 resamples × 12 sets of data). The membership probability was tested using K-fold cross-validation. Individuals from each population were divided into K groups. One of the K groups was tested by the predictive model that was built based on the remaining K-1 groups until every group was tested. In this study K was set as 3, 4, and 5. In each group, the top 10%, 25%, and 50% of high F_st_ loci as well as all loci were sampled as training data. In total, 48 assignment tests were performed for membership probability.

### Blind tests of assignment correctness

Seventeen wolves and 18 dogs were used for blind tests based on the predictive function assign.X in R that was built previously using genotype data of reference wolves and dogs^63^. The naive Bayes model was selected for the assignments. The membership probabilities of blind test samples were obtained and exhibited in a column diagram.

### Repeatability and reproducibility test

Based on our preliminary experiments, we used sufficient template input (~ 10 ng) to genotype all wolves and dogs on the same set of instruments. The success of one-time genotyping and the cumulative success of repeat-once genotyping were recorded as indicators of repeatability. Also, one-time genotyping of both dog and wolf samples from different tissues with different quality were calculated for repeatability test. Results of all 3 groups from slightly decayed, moderately decayed to highly decayed samples were included. Two wolves and 1 dog were genotyped 10 times each using the same SNaPshot protocol on three different PCR machines, 3 times on an Eppendorf Mastercycler (Eppendorf, Germany), 3 times on a SimpliAmp cycler (Applied Biosystems) and 4 times on a LifePro cycler (Bioer, China). The identity of the genotype of each sample at 12 SNP loci was recorded.

### Specificity test

Specificity tests were performed using the same SNaPshot genotyping protocol for DNA samples of 12 species including human, other carnivore species, and prey species. Each sample was genotyped twice using the same protocol and allele calling criteria as for wolf and dog. Allele signals and genotypes were recorded for each SNP site and sample.

### Sensitivity test

DNA samples of two Mongolian wolves (Wolf_#53, Wolf_#58) and a dog (Dog_#4) were arbitrarily chosen and diluted serially to reach final concentration of input DNA 400.0 ng/μl, 200.0 ng/μl, 100.0 ng/μl, 50.0 ng/μl, 25.0 ng/μl, 12.5 ng/μl, 6.3 ng/μl, 3.1 ng/μl, 1.6 ng/μl, and 0.8 ng/μl in the PCR system, and were genotyped for all loci using the same SNaPshot protocol. Positive and negative allele signals were recorded for all dilution gradients and the correctness of genotyping was controlled using the original genotype obtained previously for the three individuals.

### Ethics statement

All tissue samples including skins, muscles and semen were provided by the State Forestry and Grassland Administration Detecting Center of Wildlife of China. Blood samples of live wolves and dogs were all deposited remains of serum testing samples for health examination or medical treatments in a wolf safari park and pet hospitals. All collections were approved by animal owners with signed informed consent. The human hair samples were obtained with the informed consent of all the participants. All the experiments were carried out in accordance with the relevant guidelines and regulations of Northeast Forestry University, and the protocols were approved by Ethical Review Board of Northeast Forestry University (Approval Number: 2018001).

## Supplementary information

Supplementary file1 (PDF 400 kb)

## Data Availability

No additional data available.
