# Supplementary material for: Forensic validation of a panel of 12 SNPs for identification of Mongolian wolf and dog
Source: Sci Rep. 2020 Aug 6;10:13249. doi: 10.1038/s41598-020-70225-5 (PMC7413520; doi:10.1038/s41598-020-70225-5)
Supplement: Supplementary file 1 — Supplementary file1 (PDF 400 kb) [file 41598_2020_70225_MOESM1_ESM.pdf]

Forensic Validation of a Panel of 12 SNPs for Identification of Mongolian Wolf and Dog

Hong Hui Jiang[1], Bo Li[1,2,3,*], Yue Ma[1,2], Su Ying Bai[1,2], Thomas D. Dahmer[4], Adrian Linacre[5], Yan Chun Xu[1,2,3,*]

1. College of Wildlife and Protected Areas, Northeast Forestry University, No. 26, Hexing Road, Xiangfang District, Harbin, 150040, China
2. State Forestry and Grassland Administration Research Center of Engineering Technology for Wildlife Conservation and Utilization, No. 26, Hexing Road, Xiangfang District, Harbin, 150040, China
3. State Forestry and Grassland Administration Detecting Center of Wildlife, No. 26, Hexing Road, Xiangfang District, Harbin, 150040, China
4. Ecosystems Ltd, Hong Kong, China
5. College of Science and Engineering, Flinders University, SA 5042, Australia

* Correspondence: Bo Li, Email: libo_770206@126.com; Yan Chun Xu, Email: xu_daniel@163.com.

Hong Hui Jiang, Email: jianghh_jhh@163.com; Yue Ma, Email: dornmark@163.com; Su Ying Bai, Email: syb01@163.com; Thomas Dahmer, Email: tdahmer@pacific.net.hk; Adrian Linacre, Email: adrian.linacre@flinders.edu.au.

Table S1. Sequence information of SNP markers showing SNP sites (bracketed bold capital letters against grey), upper and lower primer regions (bold and underlined).

Name	Sequence
rs22103787	tgaggtggggcagagggagaagaagagaatcttaagcaggctngacacccagcacagagcc**caaggaggggctcaacctca**ccatcctgagatcatgctctcagctgaaatcaatagtcagaagcttaacccactgggctattcaggtgatcctcccttttctatttctaatgctccctctcctctcctttagttcaaca**y[C/T]**caggtccctagagataaaatacatgacagatagcgtaatattttgtttttgtttttagctg**tgtagaaaccaacacaccccttct**tcctatgagtttttttttttttacttttattaggaaagcaggatggagcttgagttattgtactattaatgcatgttgcattattaacntaatctttgagaaaagggagaacct
rs22835438	gggccactaacaagcttttgcaagagggcaaggcggtgttggtggaacatgtggcagccggcagcatttttcactgtctcctctaccgagctaactgctcagctg**gggattgaacccctgacctgagt**ctcattagcagtaattctgaccccaccggaccaaccaagaaacgtaggcgtcatggtatatctgtaaatcag**k[G/T]**attcggttctttgaagtcctttggttatattatggaggaacctcaa**acgaggggtggtcatggttca**gcnactatgggatactatgggtttggtggttcctcaaaaagtcaaacacggaatcgcattatgactgagcaatcacactcctgggtagacagccaaagaggtgaaaacagttatccggactgaaggtcctaca
rs23249721	gctgtatgtcaccaaggaaagagaaggcccggcggagagggacgggatccttcctaatacacaactgccatacataacgtttgcataggctgagctgataagttgtgacagatgttaa**tgaaatgctctccggctgaggt**tttcatgaacctcattaactgtcgctacttgcacattcaggaaatgcccggtggattga**y[C/T]**tgatgtttgttatctcaaattctgggtaaatgggtttttaggatgaggtgaagaaagaagatgag**ctcctgggacatttagcatgga**aaatgtatttgatagccttgccgcatgtaaaatgtgagccgtccttggtttggcacttggcaaatcttatcatatgcacttggcattagcttgcccccacccccctgctcccc
rs23608542	ttgtccgtgtgattggaaaggcagcctctccacgaatcctctcagcaggctggcagattaaaaaaaaatggagtgtgagctgagttgagcttcttgagtttcttctcccagattcatgtaacattcctcacttcttcatcaaggtgaggagagaagctagacgggctgac**acttctccacgcggaaacactt**ggagaaga**r[A/G]**aataacggctctacgctaacattttatttcaatttataaataaaatgccttggactcttatttatgatttctgggaattcctgtgtta**ggttctaatgccaccttccatttc**aattccttggtctcaccatcaggtattcctctcttaccaggctgtttcactcagccgcaggctttctccaagtcactgtatcttttgc
rs23882488	aaaacacccaaggtgattctctattttccttcttacgtataatctataacaaaactgacatcttggctgggaaaaaggattagattctcattgtttgcttcccagtattagcctttttggaaaattatagagcttctgaagacta**gtcctagtcctggtccatagaagagc**ctgaaaatagctcatttgcccagatatg**k[G/T]**aatacacaatgtttatgcacaaaaaattctgctttaggcataagatctagacacttaaaatagaatgccaaaattgcaacagacaaaaaaaggatggagcac**atctatcctccacttttctcggactt**ggtcccagcacaatatacctcaggatgtatttactgctgacggatgtacagtagcaattcatcttgcatgac
rs24163825	acatttccatgttgctcaacagtaggcctcccactgcttgtattttatgcttattgaagttcaacaaanactggagtgtctactca**gctagtcctatgccctgaaactcat**aaaattgtcctccaaggtacagaagaataagtaaagaatgacttgccagggtccgtgctggatgttttacttatattttcagtgcatat**k[G/T]**attactattctcaccaaat**aggtagcaaaactaaggctcataagga**ttaagcctgattttagagtttaagctctttatgttattctgtatcctcttaatgcaaaaatgaactcactcactcactaaaatatttaactctctaacaaacttaacaggtaatgttggtgataatgttaaaatacactttttttccccaaactc

rs24189603	gagcagccgaggaatgaatgcagtggcaacttccctcaatgcctgctaatatagacagcatttcatcagtgtgatcctccgcaacctcttgcaaggattaataatggggaataaatcacttgagggagactcagccgcaggccctagcg**ggagctcggagtgactatactggaat**tctggagaaaatgaggaaagagaaa**r[A/G]**gtgacaaacagggcta**cagagaaacagactgcatccgagg**ggccctggtggacggagggaagcagacctgagagtaggaaagcgtgggtgctggttctttcttccttgtggctcaccccatttccgcggctcctgaatccacctgcatgacggagaggctgtctccttaaactttccccttgggacataattttgaatgt
rs24198287	atggcaacccctacctaagtctattg**gtaggccagtcttcagtgtggtga**cactgcactcacacagcatagcattctatacatgctatttgtatttgaaatatgatgttttgctccatattttccttctaaagtatgcttttcttaagctgttgttaaaaaaaaaagttcgccttcatcagttacaaataattgtggtg**r[A/G]**cccaagcacatggtaagtgacagtgaagggcnattctacttgcagaaactcaagtgctaactttattaggagaaaaaaaaaattgcaaatgaatttagcatgagtgtgaagtactgacaactaaaa**gaaaaccaccaggaatcatgcc**aagtaatgattttagnttcaagttacaatgacctactttgtggtttctcaac
rs24355642	ttaccaccactcaagtctatntctctaccttttctacttttctctacttctaacttatatggccaaggatcttatatactttggagctaactaactatggcattttaaagaacaaaattgtaactctaaatattagctttatagcatttacagatcatattcctatcttttgca**ctcataaaatcgggagccttgctt**aa**y[C/T]**gtagactaattaaacgaagggaagttactgtcaaggttaagaatctgaaaaat**tctgctggattgctgtgtatactgc**tgaatgagtcattaaaacagtagttttcagagtgcaatctttgaaccagcatttttggcatgatcaatganctttttagaaatataaattcataggccctaccttaacctagatgtgcctag
rs24383001	cttcactaattccaaacttggcgagatcccacagagctgttcactccacggtccctctctgactcatctcatttattttcctgtgggagaaataaggcgcagatagataaacgatgccacccaaggttccccagccagggagtggcagagagggtgttcaaactcatt**tacgtctgacgccaaaacccact**gcaacacac**y[C/T]**gctcttcattctcggttccaagactaaattcaggaaccaaaaataacagaacagctgtgtgtttgc**agtggcccaaatgtgcaggtg**cacgttgagcctgagccatatttccaccttgattctgatnttgaagtttctattaggaacacccaaatgactgcctctttcttcttgctaaaaaatatacatgatgtatatat
rs24863098	gggctggtttaagaccttacccctttagcagactcatatttttatgagccccatccatgtccaatcaaacctcccaaatgcaccttgcaccttactgaaggcatttgtgttttactact**gtgagcagagttgcatggcaca**cactgacatattcttagctgggtagatatttgacggagtgcataatgatccctgtgtac**m[A/C]**gcattcctcccttagtttggagctgaggatcccctccttggtcc**tcgtccttgagaggcagcagc**tgggtaagttctctgagtttgaccttcacagacaggaaggctcccatgcaggttctagttaccactgatcagtgtctaaaatgtagataagcttagtgcaccaggaaaatttatggaacagtggcgaatagaaac
rs9089629	caaactttgcctttgttttttctggaaagattgctgtgctctaagtgaaagcttgtagaatttaccagaagaaatgattgcattaagtaggccatttcttgggtt**tggatttgttgcctctcccctagt**aaaggcaatttactacttaaccttcatgtttatgtgcccatgaagccaaccactggattactgccattga**r[A/G]**attcaaatttgggagaggggaacat**ggatgtagctgcccatttacagtgtag**ggttcattataagattacctcaaggttttaatgactttctattgtaaaaatcaatgtgtccattgtagaaaatttggaaactactaaaaggagttaaagaaaaataacacctgcaatcacatctcccagagataattatcaacatttt
rs9159232	cccctcagtaagtcttctgccttgcactgcaccttcccttgcatgatgggacctgcaacgacactagatttgctgtgaa

gatgctcatataaagaatagaatccc**tcccacctacatctgcttctgtgt**ttttctccaagcacagctgagcactttg
cttttatttccagatgcaggtggccaggcctgcatggcagaga**r[A/G]**gaaactaggtcacagggacgccgta
ggtcacctacagggagacccga**gtctcagttccttgacaagtcatccc**caggtgagaccccgtccagtgccac
acacagcgtgtgtgcataggaagtgggtgatccagtccagatcagtggccagccttctttgatttccagtcccctagt
tttaccacgatgtcaacctcatgc

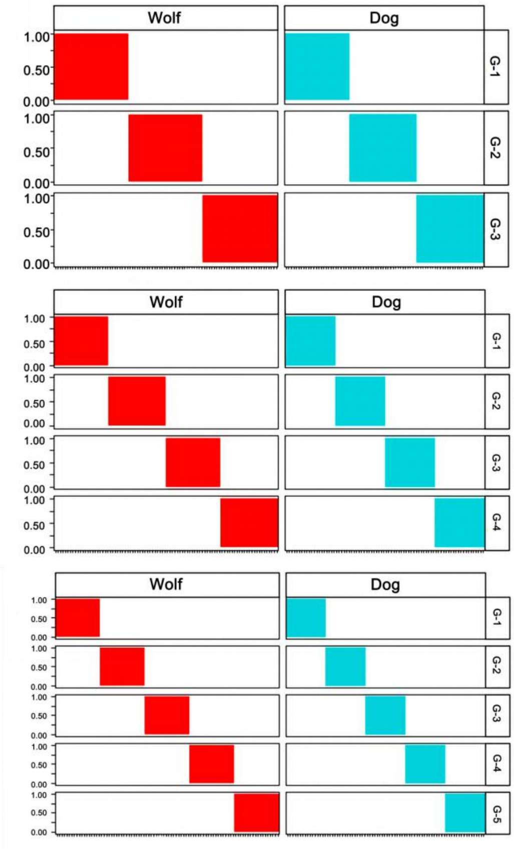

Fig. S1. Membership probability of two hypothetical populations of Mongolian wolf (red) and domestic dog (blue) with results estimated via 3, 4 and 5-fold cross-validation using all 12 SNP loci.
